# Protection against canine parvovirus type 2 infection in puppies by colostrum-derived antibodies

**DOI:** 10.1017/jns.2014.57

**Published:** 2014-11-13

**Authors:** Hanna Mila, Aurélien Grellet, Costantina Desario, Alexandre Feugier, Nicola Decaro, Canio Buonavoglia, Sylvie Chastant-Maillard

**Affiliations:** 1 Université de Toulouse, INP, ENVT, UMR 1225, IHAP, F-31076 ToulouseFrance; 2 INRA, UMR1225, IHAP, F-31076 Toulouse, France; 3 Royal Canin, 650 Avenue de la Petite Camargue, Aimargues, France; 4 Department of Animal Health and Well-Being, Faculty of Veterinary Medicine, Bari University, Bari, Italy

**Keywords:** Puppies, Canine parvovirus, Colostrum, Maternally derived antibodies, CPV2, canine parvovirus type 2, HI, haemagglutination inhibition, MDA, maternally derived antibodies

## Abstract

During the first weeks of life puppies remain protected against canine parvovirus type 2 (CPV2) infection thanks to maternally derived antibodies (MDA) absorbed with colostrum after birth. The objective of the present study was to present the variability in CPV2-specific passive immune transfer and its consequences in puppies naturally exposed to the parvovirus. Seventy-nine puppies from one breeding kennel were included in the study at birth and followed until 56 d of age. Once per week the MDA titre for CPV2 specific antibodies was determined in blood. Viral excretion was also evaluated on a rectal swab by CPV2 PCR assay and puppies were weighed to determine growth rate. At 2 d of age, thirty-four out of seventy-nine puppies (43 %) had MDA ≤1:160 (designed group A) and forty-five puppies (57 %) had greater MDA titres (designed group B). The level of absorbed maternal antibodies was shown to be associated with breed size and growth rate during the first 48 h of life. The MDA level declined with age in all cases; however, the proportion of puppies with the antibody level considered as protective against CPV2 infection was significantly higher in group B compared with A from day 2 until 42. Among all puppies surviving until 56 d of age, sixty-seven out of seventy (95·7 %) underwent CPV2 infection. However, puppies from group A excreted CPV2 significantly earlier than puppies from group B. The present study demonstrates the link between passive immune transfer, in terms of level of specific MDA absorbed, and length of the protection period against parvovirus infection in weaning puppies.

The prevalence of canine parvovirus type 2 (CPV2) in diarrheic puppies varies from 64 % in North America and 70 % in Europe^(^^1^^,^^2^^)^. CPV2 is a ubiquitous enteropathogen that is responsible for outbreaks of acute gastroenteritis, with a high mortality rate^(^^3^^)^. During the first weeks of life, maternally derived antibodies (MDA) provide the only specific systemic protection against CPV2 in puppies. Only 10 % of circulating CPV2 antibodies in the neonate are from transplacental origin^(^^4^^)^. The vast majority is transferred from the dam to puppies through colostral ingestion during the first hours of life. Systemic CPV2 MDA titre decreases with age^(^^4^^–^^6^^)^. When the serological titre has fallen under 1:80 (haemagglutination inhibition (HI)), the systemic MDA level seems to be no longer protective against CPV2^(^^4^^,^^7^^)^. To date, the variability in maternally derived protection against CPV2 and its consequences in puppies have been studied exclusively under experimental conditions and only on weaned puppies or puppies deprived from maternal milk. Since canine colostrum and milk have been proven to provide significant amounts of CPV2 antibodies^(^^6^^)^, these lactogenic MDA could potentially interfere with CPV2 intestinal replication either by coating the enterocytes or trapping the faecal CPV2 particles, preventing their multiplication in the mucosa. Moreover, since viral challenge induces a more rapid decrease in circulating CPV2 antibodies,^(^^8^^)^ MDA kinetics may differ depending on environmental infective pressure.

In the present study, we focused on CPV2 MDA in puppies maintained under natural conditions, housed in a breeding kennel with natural CPV2 circulation. The aim of the work was to analyse the variability and kinetics of systemic maternally derived CPV2 antibodies under these field conditions and to evaluate factors influencing MDA. The link between initial MDA level, viral shedding and growth performance was also studied.

## Materials and methods

The study protocol was reviewed and approved by the Royal Canin Internal Ethics Committee.

### Animals

The experiment was conducted in a commercial breeding kennel over a 4 months period (March–June 2012). Seventy-nine puppies from various breeds (twenty-six litters, ranging from one to eight puppies per litter alive at day 2; day 0 = whelping) were included and followed from birth until 56 d of age. Breeds whose adult weight was less than 25 kg were considered small breeds and dogs with a greater adult weight large breed dogs. All puppies were housed with their dam in heated whelping boxes from birth to 56 d. Puppies were allowed to suckle freely. Lactating bitches and their puppies were fed *ad libitum* with the same diet, a dry expanded complete diet balanced for growing dogs (Starter, Royal Canin).

Blood (1 ml per puppy) was collected from the jugular vein at days 2 and 7 and every week until day 56. Samples were immediately centrifuged (3000 ***g***, 15 min) and serum separated. Rectal swabs were performed at day 17 and every week until day 52. Sera and rectal swabs were stored for 4 months at −20°C until assayed.

### Canine parvovirus type 2 antibodies assay

Titres of antibodies directed against CPV2 were evaluated on serum by HI test as previously described^(^^7^^)^. Tests were performed at +4°C using ten haemagglutinating units of CPV2 antigen and 1 % pig erythrocytes. All samples from one puppy (from one to nine samples) were tested on one plate with a maximum of ten plates performed at one time. Twofold dilutions in PBS of each serum sample starting from 1:10 were tested and the HI titre was the highest serum dilution completely inhibiting viral haemagglutination. Titres below 1:80 were considered as non-protective against infection^(^^4^^)^. Seroconversion was defined as a minimum 4-fold increase of HI titre. After a seroconversion episode (indicating a viral contamination), puppies were considered as no longer protected by MDA.

### Canine parvovirus type 2 faecal excretion

A homogenate (10 %) of the faecal sample was prepared in PBS (pH 7·2) and centrifuged at 1500 ***g*** for 15 min. The viral DNA was extracted from prepared supernatant by boiling the sample (10 min) and subsequently chilling on ice. To reduce inhibition of DNA polymerase, samples were diluted 1:10 with distilled water. No more than ten extractions were performed at one time. CPV2 real-time PCR assay with the TaqMan probe was conducted on faecal samples as described by Decaro *et al.*^(^^9^^)^ with ovine herpesvirus 2 DNA as internal control. A dilution of standard DNA in a CPV-negative faecal suspension was performed (serial log 10 dilutions) and tested to determine the detectability and the linearity of the assay. The following thermal protocol was used: iTaq DNA polymerase was activated at 95°C for 10 min followed by forty cycles consisting of denaturation at 95°C for 15 s, subsequently primer was annealed at 52°C for 30 s and the process was extended at 60°C for 1 min.

### Growth

Puppies were weighed at birth, 48 h and every week until 56 d of age using a calibrated analytical scale in 1 g increments (Fisher Scientific International Inc.). Subsequently, growth rate (%) over the first 2 d of life was calculated ((weight at 2 d – weight at birth)/weight at birth × 100), together with growth rate between 21 and 56 d of life ((weight at 56 d–weight at 21 d)/weight at 21 d × 100).

### Statistical analysis

Statistical analyses were performed using Tanagra^®^ freeware (Tanagra 1·4, Lyon, France). All datasets were tested for normality by the Shapiro–Wilk test. As data were not normally distributed, they were presented as medians and range. A two-sided Mann–Whitney *U* test or a Kruskal–Wallis test was used according to the number of groups considered. The level of statistical significance was set at *P* < 0·05 for all analyses.

## Results

### Variability in canine parvovirus type 2-specific passive immune transfer

**Fig. 1.**
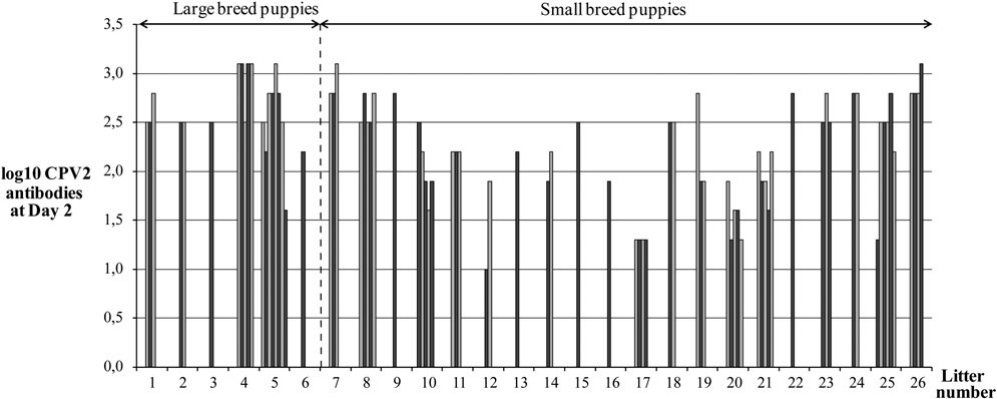
Variability in maternally derived antibody titres against canine parvovirus type 2 at 2 d of age (*n* 79). Each bar represents one puppy, each group of bars represents one litter (*x*-axis), and puppies are divided in small and large breeds (arrows).

At 2 d of age, MDA titres displayed large variability between puppies, titres ranging from 1:10 to 1:1280 (log10 = 1–3·1; Fig. 1). At that time (day 2), thirty-four out of seventy-nine puppies (43 %) had HI titre ≤1:160 (log10 ≤ 2·2; group A), among which thirteen (38 % of total population) had not reached the HI titre 1:80 (log10 = 1·9), considered as the minimal protection against CPV2 infection. Only forty-five of seventy-nine animals (57 %) had HI titres > 1:160 (group B) with seven puppies at HI ≥ 1:1280. Mortality rate between 2 and 56 d of age was significantly higher in puppies from group A than group B (9/34; 26 % *v.* 3/45; 7 %; *P* = 0·022).

### Factors influencing canine parvovirus type 2-specific passive immune transfer

The breed size and growth rate between birth and 48 h of life were associated with CPV2 specific antibody transfer from bitch colostrum to puppies. Large breed puppies had higher MDA titres at day 2 compared with small breed puppies (median HI titre: 1:320 (range: 1:40–1:1280) *v.* 1:160 (1:10–1:1280); *P* = 0·003). Puppies which lost weight during the first 48 h of life had lesser MDA titres at day 2 than puppies which gained weight (HI titre: 1:120 (1:10–1:1280) *v.* 1:320 (1:40–1:1280); *P* < 0·001).

### Maternally derived antibody level and canine parvovirus type 2 infection

#### Kinetics of canine parvovirus type 2 maternally derived antibodies

The titres of MDA progressively declined with age in both groups. The proportion of puppies with MDA protection against CPV2 infection in group B was significantly higher from day 2 until 42 than in group A. At day 56 none of the 67 surviving puppies displayed MDA titre ≥1:80 (Table 1).

**Table 1.** Proportion of puppies protected from CPV2 infection (HI ≥ 1:80) depending on MDA level at 2 d of age

	Age of puppies (weeks)								
	2	7	14	21	28	35	42	49	56
Group A	21/34 (62)	14/30 (47)	5/26 (19)	0/26 (0)	0/25 (0)	0/25 (0)	0/25 (0)	0/25 (0)	0/25 (0)
Group B	45/45 (100)	44/44 (100)	41/44 (93)	34/44 (77)	24/42 (57)	10/44 (23)	9/44 (20)	2/44 (5)	0/43 (0)
*P*-value for each period of time	<0·001	<0·001	<0·001	<0·001	<0·001	0·011	0·021	0·531	–

*n*_i_/*n* = number of puppies protected in the category considered/total number of puppies in the category (%).

Sixty-three out of the sixty-seven puppies still alive at the end of the experiment underwent a seroconversion, with sixty-two of them reaching an HI titre of 1:1280. The four puppies which did not seroconvert belonged to group B. Median age at seroconversion was 49 d (28–56) in puppies from group A and 56 d (35–56) in group B (*P* < 0·001). Among thirteen puppies with MDA titre at day 2 < 1:80, the first seroconversion appeared at day 42. Half-life of systemic MDA was 13·4 d.

#### Viral excretion

Among seventy puppies still alive at 17 d of age, sixty-seven (96 %) displayed a significant viral excretion (≥1000 copies) at some point during the study period. Puppies from group A excreted CPV2 at significantly earlier age compared with puppies from group B (day 38 (17–52) *v.* day 45 (17–52); *P* = 0·011).

At the time of the first significant viral excretion, fifty-two puppies had HI titres less than 1:80, fourteen puppies had HI titres of 1:80 or 1:160 and only three puppies had HI titres > 1:160.

#### Growth

Growth rates between 21 and 56 d of age were not significantly different between groups A and B (65 % (53–83 %) *v*. 62 % (28–79 %); *P* = 0·11).

## Discussion

MDA are crucial for the protection of puppies against CPV2 infection since puppies are nearly agammaglobulinemic at birth. Canine neonates acquire systemic antibodies via colostral ingestion within the first hours of life before gut closure^(^^10^^)^. In the present study, at 2 d of age, MDA titres displayed large variability between puppies, with titres ranging from 1:10 to 1:1280. The variability in MDA level could be due to unequal colostrum ingestion from maternal or puppy's origin. In the present study, we noted a relationship between the early growth rate and the absorption of specific CPV2 MDA. Both reflect colostrum intake as this secretion plays not only an immune, but also a nutritional role. Systematic weighing of puppies could therefore be performed by breeders in order to control for correct passive immune transfer and energy intake at the very early stages of life.

After the first 24 h of life, MDA are no longer absorbed and they decline with age^(^^4^^,^^6^^)^. Pollock & Carmichael^(^^4^^)^ observed a half-life for CPV2 MDA of 9·7 d, with puppies reaching seronegative levels between 10 and 14 weeks of age. Gooding & Robinson^(^^5^^)^ observed the HI titres <1:10 after day 49. In the present study, half-life was slightly longer at 13·4 d. From the observation of Macartney *et al.*^(^^8^^)^, who described an acceleration in the decline of blood CPV2 titres after viral challenge, one could expect a more rapid MDA decrease in the present study, which was conducted under a high CPV2 environmental pressure. In this situation, systemic MDA may be recruited to limit the multiplication of CPV2 virus, thereby leading to an earlier entry into a susceptibility period for viral infection. Nevertheless, in our conditions of natural infection, this hypothesis was not confirmed.

The early consumption of a sufficient quantity of maternal colostrum to maximise passive immune transfer appears to increase the length of the protective period. Indeed, the proportion of protected puppies was higher in the group with the higher MDA level (group B) until 42 d of age. The study demonstrates thus the importance of optimal colostrum intake in puppies in order to induce a longer immunoprotection during the pediatric period.

The large variation in the CPV2 susceptibility period between puppies observed in the present study underlines that a routine vaccination protocol should be adapted not only to the breeding kennel epidemiologic situation, but also to a puppy's individual needs. Although the early vaccination appears controversial since MDA may interfere with CPV vaccination, decreasing the vaccines response^(^^4^^,^^11^^,^^12^^)^, recently a high antigen titre vaccine administrated as early as 4 weeks of age was demonstrated effective in the reduction of the CPV2 susceptibility window^(^^13^^)^.

Over the study period, nearly all puppies (96 %) underwent viral infection and seroconversion. Viral infection appeared in the vast majority of puppies when HI titres were lower or equivalent to 1:80 (90 % of the infected puppies with HI ≤ 1:80), as described for experimental viral challenges^(^^4^^,^^7^^)^. In puppies with a MDA level lower than protective against CPV2 infection at birth, seroconversion appeared for the first time only at day 42. Further work would be necessary to assay pathogenic viral loads (by haemagglutination test) in parallel with global viral load (as obtained by PCR), together with coproantibodies^(^^11^^)^, to verify the importance of lactogenic MDA before and during natural CPV2 infection episodes.

### 

#### Conclusions

Based on systemic MDA, optimal passive immune transfer lengthens the protection period against CPV2 infection. Breeders should be encouraged to pay attention to early suckling within the first 12 h after birth. Systematic weighing at early age, evaluating indirectly the passive immune transfer, could indicate puppies at risk and allow adaptation of the vaccination protocol to individual needs. Nevertheless, the potential role of local MDA, as provided by milk, to limit viral replication and its consequences on morbidity and mortality merits further investigation.
